# Soluble VCAM-1 impairs human brain endothelial barrier integrity via integrin α-4-transduced outside-in signalling

**DOI:** 10.1007/s00401-015-1417-0

**Published:** 2015-03-27

**Authors:** Axel Haarmann, Eva Nowak, Annika Deiß, Susanne van der Pol, Camelia-Maria Monoranu, Gijs Kooij, Nora Müller, Paul van der Valk, Guido Stoll, Helga E. de Vries, Friederike Berberich-Siebelt, Mathias Buttmann

**Affiliations:** 1 Department of Neurology, University of Würzburg, Josef-Schneider-Str. 11, 97080 Würzburg, Germany; 2 Department of Molecular Cell Biology and Immunology, MS Center Amsterdam, VU University Medical Center, Amsterdam, The Netherlands; 3 Department of Neuropathology, University of Würzburg, Josef-Schneider-Str. 2, 97080 Würzburg, Germany; 4 Institute of Virology and Immunobiology, University of Würzburg, Versbacher Str. 7, 97078 Würzburg, Germany; 5 Department of Pathology, Neuroscience Campus Amsterdam, VU University Medical Center, Amsterdam, The Netherlands; 6 Department of Molecular Pathology, University of Würzburg, Josef-Schneider-Str. 2, 97080 Würzburg, Germany

**Keywords:** Multiple sclerosis, Blood–brain barrier, Endothelial cell, Integrin alpha4, Vascular cell adhesion molecule-1, Natalizumab

## Abstract

Human brain microvascular endothelial cells forming the blood–brain barrier (BBB) release soluble vascular cell adhesion molecule-1 (sVCAM-1) under inflammatory conditions. Furthermore, sVCAM-1 serum levels in untreated patients with multiple sclerosis (MS) correlate with a breakdown of the BBB as measured by gadolinium-enhanced MRI. To date, it is unknown whether sVCAM-1 itself modulates BBB permeability. Here, we provide evidence that human brain endothelium expresses integrin α-4/β-1, the molecular binding partner of sVCAM-1, and that sVCAM-1 directly impairs BBB function by inducing intracellular signalling events through integrin α-4. Primary human brain microvascular endothelial cells showed low to moderate integrin α-4 and strong β-1 but no definite β-7 expression in vitro and in situ. Increased brain endothelial integrin α-4 expression was observed in active MS lesions in situ and after angiogenic stimulation in vitro. Exposure of cultured primary brain endothelial cells to recombinant sVCAM-1 significantly increased their permeability to the soluble tracer dextran, which was paralleled by formation of actin stress fibres and reduced staining of tight junction-associated molecules. Soluble VCAM-1 was also found to activate Rho GTPase and p38 MAP kinase. Chemical inhibition of these signalling pathways partially prevented sVCAM-1-induced changes of tight junction arrangement. Importantly, natalizumab, a neutralising recombinant monoclonal antibody against integrin α-4 approved for the treatment of patients with relapsing–remitting MS, partially antagonised the barrier-disturbing effect of sVCAM-1. In summary, we newly characterised sVCAM-1 as a compromising factor of brain endothelial barrier function that may be partially blocked by the MS therapeutic natalizumab.

## Introduction

Cell-bound vascular cell adhesion molecule-1 (VCAM-1, CD106) allows human brain microvascular endothelium to control immune cell trafficking across the blood–brain barrier (BBB). It is upregulated in inflammatory-active brain lesions of patients with multiple sclerosis (MS), a chronic degenerative autoimmune disease of the CNS [1, 16, 45]. Endothelial VCAM-1 serves as a binding partner for integrin α-4/β-1 (very late antigen-4, VLA-4) on peripheral blood mononuclear cells (PBMC), as it does for α-4/β-7 heterodimers to a lesser extent [5, 17]. This molecular interaction enables PBMC to firmly adhere to the vessel wall after rapid activation of integrin α-4-mediated intracellular signalling cascades, allowing subsequent immune cell extravasation [39].

A soluble form of VCAM-1 (sVCAM-1) is shedded from the surface of brain endothelial cells upon inflammatory activation [24]. In vitro, sVCAM-1 blocked leukocyte adhesion to activated human brain endothelium. Soluble VCAM-1 was therefore considered as an inflammation-limiting factor at the inflamed BBB [25]. Clinical studies in MS patients treated with recombinant interferon-β (IFN-β), which moderately reduces relapse rate, disability progression and MRI disease activity [9], seemed to support this hypothesis: IFN-β therapy increased sVCAM-1 serum levels, which correlated with a reduction of gadolinium (Gd)-enhancing MRI brain lesions, indicating less inflammatory disease activity at the BBB [12, 20, 38]. Together, these in vitro and clinical studies suggested a local anti-inflammatory effect of sVCAM-1 at the human BBB due to an inhibition of immune cell extravasation [14].

In addition to their regulation of immune cell trafficking, brain endothelial cells strictly govern the exchange of solute and soluble factors across the BBB. Endothelial molecular control mechanisms include active transendothelial transport systems and tight junctions. The latter are highly dynamic trans-membrane protein complexes, tightly sealing the interendothelial clefts [46]. Extravasation of soluble factors such as albumin or immunoglobulins is a direct correlate of BBB dysfunction as visualised by Gd-enhanced MR imaging [23, 30]. Interestingly, sVCAM-1 serum levels in MS patients not receiving IFN-β treatment were shown to positively correlate with the presence of Gd-enhancing lesions on brain MRI scans and with clinical disease activity in a majority of studies [15, 18, 19, 22, 36, 37]. This seems to contradict the IFN-β studies cited above where an inverse correlation between sVCAM-1 serum levels and MRI disease activity was observed. The mechanisms underlying these divergent findings are unknown. Furthermore, it currently remains unknown whether sVCAM-1 exerts any direct effect on brain endothelial barrier function.

So far, it is unknown whether undiseased human brain endothelial cells or those in MS CNS lesions express the established binding partners of sVCAM-1, i.e. integrin α-4 heterodimers. An expression of integrin α-4 by human brain endothelium in situ was previously described in activated glioma endothelial cells [33]. Furthermore, expression of integrin α-4/β-1 and of α-4/β-7 was reported in human umbilical vein endothelial cells (HUVEC) and in adult human synovial membrane endothelium from patients with rheumatoid arthritis [6, 29]. VLA-4 expression was furthermore documented in adult human dermal microvascular endothelial cells [26]. Endothelial integrin α-4 expression was lower than integrin β-1 expression in these endothelial cell types, but could be upregulated by pro-inflammatory stimulation with TNF-α [6, 32]. HUVEC binding to recombinant VCAM-1 and to the extracellular matrix protein fibronectin was found to be mediated by VLA-4 [26, 29]. Furthermore, sVCAM-1 was shown to activate p38 MAP kinase and focal adhesion kinase in HUVEC via integrin α-4, which promoted HUVEC migration in vitro and angiogenesis in mouse corneas in vivo [32]. Effects of sVCAM-1 on the barrier function of integrin α-4-positive endothelial cells have not been reported to date.

Here, we investigated the expression of sVCAM-1-binding integrins on adult human brain endothelium both under non-inflammatory and inflammatory conditions, and studied whether and how sVCAM-1 affects the barrier function of cultured primary human brain microvascular endothelium.

## Materials and methods

### Brain tissue

Cryopreserved early post-mortem autopsy specimens from normal human brain and spinal cord of three donors who had died from non-neurological diseases and cryopreserved brain biopsy samples from two donors without pathological changes in the biopsy material used for this study were from the brain bank at the Department of Neuropathology in Würzburg and used for research purposes as approved by the local Ethics Committee of the Faculty of Medicine at the University of Würzburg.

Furthermore, brain tissue from seven cases with clinically diagnosed and pathologically confirmed MS (3 female, median disease duration 22 years, median age at death 60 years, range 43–90) and four non-neurological control cases (2 female, median age at death 82 years, range 73–91) was obtained by rapid autopsy (median post-mortem delay 6 h, range 4–18 h) and immediately frozen in liquid nitrogen (The Netherlands Brain Bank, Amsterdam, coordinator Dr. Huitinga). The Netherlands Brain Bank received permission to perform autopsies for the use of tissue and for access to medical records for research purposes from the Ethical Committee of the VU University Medical Centre, Amsterdam, The Netherlands. Supratentorial white matter MS and control tissue samples were selected on the basis of post-mortem MRI. All patients and controls, or their next of kin, had given informed consent for autopsy and use of their brain tissue for research purposes. MS lesion activity was classified based on the expression of myelin and inflammatory cells, defined by staining for proteolipid protein and HLA-DR as described before [13, 27].

### Brain endothelial cells

Cryopreserved single-donor primary human brain microvascular endothelial cells (HBMEC) isolated from normal human brain and reported to be mycoplasma negative by the provider were purchased from Cell Systems Corp. (Kirkland, WA, USA) at passage 2. Preparations were analysed from nine different donors. Before experimental use, each preparation was extensively characterised for potential contamination by other cell types and for expression of tight junction-associated molecules as previously described [8]. The endothelial cell fraction was >98 % in all samples. A well-characterised immortalised HBMEC line (HBMEC_Kim_) was kindly provided by Kim [43]. Both cell types were plated on culture dishes (Nunc, Roskilde, Denmark) coated as indicated and cultured at 37 °C/5 % CO_2_ in Medium 199 (Lonza, Cologne, Germany) containing 10 % foetal calf serum (FCS; Biochrom, Berlin, Germany), endothelial cell growth supplement (20 μg/mL; Sigma-Aldrich, Schnelldorf, Germany), heparin (100 μg/mL; Sigma-Aldrich), amphotericin B (250 μg/mL), gentamycin (50 μg/mL), penicillin (50 U/mL) and streptomycin (50 μg/mL; all from Invitrogen, Karlsruhe, Germany). All primary cell preparations as well as HBMEC were mycoplasma negative, as revealed by a commercial PCR-based mycoplasma detection kit (PK-CA91; PromoKine, Heidelberg, Germany). Primary endothelial cells were used from passage 4 to 6 for experiments, immortalised endothelial cells from passage 20 to 30.

### Immunohistochemistry

For immunofluorescence histochemistry, 5-µm cryostat sections were blocked with a donkey serum solution and incubated with primary antibodies (Abs) at 4 °C overnight. Tested monoclonal mouse Abs against integrin α-4 included clones 2B4, 7.2R (both from R&D, Karlsruhe, Germany) and L25 (BD Biosciences, Heidelberg, Germany). For staining of integrin β-1, we used a rat monoclonal Ab (clone mAb13, BD Biosciences), and for integrin β-7, we employed the mouse monoclonal Ab FIB504 (BD Biosciences). Rabbit polyclonal Abs against von Willebrand factor (vWF; DAKO, Hamburg, Germany) or laminin (MP Biomedicals, Eindhoven, Netherlands) were used to stain the brain vasculature. A biotinylated antibody against HLA-DR (clone LN3) was used to stain immune cells. Isotype-matched control Abs were from BD Biosciences. Fluorescence-conjugated secondary Abs were from Dianova (Hamburg, Germany) and incubated for 1 h.

For bright field stainings, sections were deparaffinised and treated with 0.3 % H_2_O_2_ in methanol for 20 min to reduce endogenous peroxidase activity. Antigen retrieval was achieved by incubating the sections at 100 °C in Tris–EDTA buffer (10–0.5 mM, pH 9.0) for 10 min. After washing with phosphate-buffered saline (PBS), sections were treated with 0.1 % saponin in PBS, washed, and subsequently incubated with the same primary Abs as used for immunofluorescence histochemistry in PBS overnight at 4 °C. Slides were then washed and incubated with EnVision + Dual Link reagent (DAKO, Glostrup, Denmark) for 30 min followed by visualisation with the peroxidase substrate diaminobenzidine (DAB) (DAKO, Glostrup, Denmark). After a short rinse in tap water, sections were incubated with haematoxylin for 1 min and extensively washed with tap water for 10 min. Finally, sections were dehydrated with ethanol followed by xylene and mounted with Entellan (Merck, Darmstadt, Germany).

Samples were analysed with an Olympus IX-70 inverted system microscope with IX-FLA observation attachment for fluorescence imaging, with a Zeiss LSM 780 confocal microscope or with a Leica DM6000 microscope (Leica Microsystems, Heidelberg, Germany).

### Flow cytometry

HBMEC were grown to subconfluency on 6-well plates and then stimulated as indicated. Vascular endothelial growth factor-165 (VEGF_165_) was purchased from PeproTech Inc. (Hamburg, Germany) and TNF-α was from R&D Systems. Thereafter, cells were detached with 500 μL Accutase™ (PAA Laboratories, Coelbe, Germany), washed and incubated with uncoupled mouse anti-integrin α-4 (clone L25), PE-coupled mouse anti-integrin β-1 (clone MAR4, BD Biosciences), PE-coupled mouse anti-integrin β-7 (clone FIB504, BD Biosciences) or isotype-matched control Abs (all from BD Biosciences) for 30 min at 4 °C. For integrin α-4 staining, cells were additionally incubated with a FITC-labelled secondary Ab against mouse IgG (R&D, Karlsruhe, Germany) after a washing step. Fluorescence-activated cell sorting analysis was performed on a FACSCalibur (BD Biosciences). For all integrin stainings including integrin β-7, human PBMC, freshly isolated by Ficoll gradient centrifugation according to a standard protocol, were used as a positive control.

### Boyden chamber assay

For paracellular permeability assays, 1 × 10^5^ per well cells were seeded on rat collagen-coated (100 µg/mL) filters (0.4 µm pore size) of a 24-well Boyden chamber system (Corning Life Sciences, Wiesbaden, Germany) and grown to confluency which usually took 3 days. Confluency was assessed by DAPI staining of filters grown in parallel. Part of the cells was pre-incubated with natalizumab (Biogen Idec, Ismaning, Germany) or a corresponding IgG4κ isotype control (Sigma-Aldrich) for 1 h as indicated. Stimulations were performed with recombinant human VCAM-1 or ICAM-1 (R&D Systems) or TNF-α plus interferon-γ (IFN-γ, R&D Systems) for the indicated durations. After stimulation, medium was removed in the upper and lower chambers and replaced by HEPES buffer. To trace cell permeability, 1 mg/mL FITC-dextran 3000 was added to the upper chambers and relative fluorescence in the lower chambers was measured 90 min later, using a Fluoroskan Ascent^®^ (Thermo Electron Corporation, Dreieich, Germany) microplate fluorometer. Dextran concentrations in the lower chambers were determined using a dextran standard curve. To maximise assay precision, experiments were performed in 12 wells per stimulation condition.

### Immunocytochemistry

For immunocytochemical stainings of tight junction-associated molecules, cells were grown on 2 % gelatin-coated 24-well plates and stimulated as indicated. Rho-associated kinases inhibitor Y-27632 and p38 inhibitor SB203580 were from Calbiochem—Merck4Biosciences (Darmstadt, Germany). Then cells were fixed with 3.7 % formalin for 10 min, permeabilised with 0.1 % Triton X-100 for 6 min, and washed with Dulbecco’s PBS. Unspecific Ab binding was blocked by 5 % BSA (Sigma-Aldrich) in PBS for 60 min at room temperature. Subsequently, primary Abs to zonula occludens (ZO)-1 (rabbit polyclonal, Invitrogen), occludin (mouse monoclonal, clone OC-3F10, Invitrogen) and junctional adhesion molecule (JAM)-A (mouse monoclonal, clone M.Ab.F11, AbD Serotec, Düsseldorf, Germany) were incubated for 60 min at room temperature. After washing, an appropriate Cy3-coupled secondary Ab was incubated for another hour at room temperature. After a further washing step, nuclei were counterstained with DAPI. After a final washing step, an antifading agent (Dabco, Merck, Germany) was added. Negative controls were performed by omitting the primary Abs and by stainings with isotype-matched control Abs (data not shown). The stainings were analysed by an Olympus IX-70 inverted system microscope with IX-FLA observation attachment for fluorescence imaging. All analyses were performed by blinded observers directly at the microscope and not from electronic images.

### Western blotting

For generation of whole cell protein extracts, cells grown to subconfluency in 25 cm^2^ flasks were stimulated as indicated, washed with icecold PBS and scraped into radioimmunoprecipitation assay (RIPA) buffer composed of 50 mM HEPES, 125 mM NaCl, 1 % Nonidet P40, 1 mM EDTA pH 7.4 and 1 × Roche COMPLETE^®^ protease inhibitor mix (Roche Diagnostics GmbH, Mannheim, Germany). Samples were shaken vigorously for 30 min, and then centrifuged at 18,000*g* for 15 min at 4 °C. Supernatants were subjected to Western blot analysis. Primary Abs were used against phospho-p38 (cat.-no. 9211, Cell Signaling, Danvers, MA, USA), phospho-ERK (cat.-no. sc-7383, Santa Cruz, Heidelberg, Germany) or phospho-JNK (cat.-no. sc-6254, Santa Cruz). Appropriate peroxidase-coupled secondary Abs were employed with a standard enhanced chemoluminescence system (Amersham, Arlington Heights, IL, USA). After peroxidase inactivation, membranes were reprobed with Abs against total p38 (cat.-no. 9212, Cell Signaling), ERK (cat.-no. sc-94, Santa Cruz) or JNK (cat.-no. sc-474, Santa Cruz).

### Rho activation assay

Rho activation assays were performed using the Rho Activation Assay Kit from Millipore (Schwalbach/Ts., Germany) according to the instructions of the manufacturer. In brief, active, GTP-bound Rho was isolated from cell extracts using a GST-tagged fusion protein corresponding to residues 7–89 of mouse Rhotekin rho-binding domain and bound to glutathione–agarose, and subsequently detected by immunoblot analysis using anti-Rho.

### Statistical analysis

For statistical analysis of the dextran permeability assays, a Kruskal–Wallis test was followed by Dunn’s post test for multiple comparisons. Calculations were performed with GraphPad PRISM 4 software (GraphPad Software, La Jolla, CA).

## Results

### Low to moderate normal brain endothelial integrin α-4 expression in situ and in vitro

**Fig. 1:**
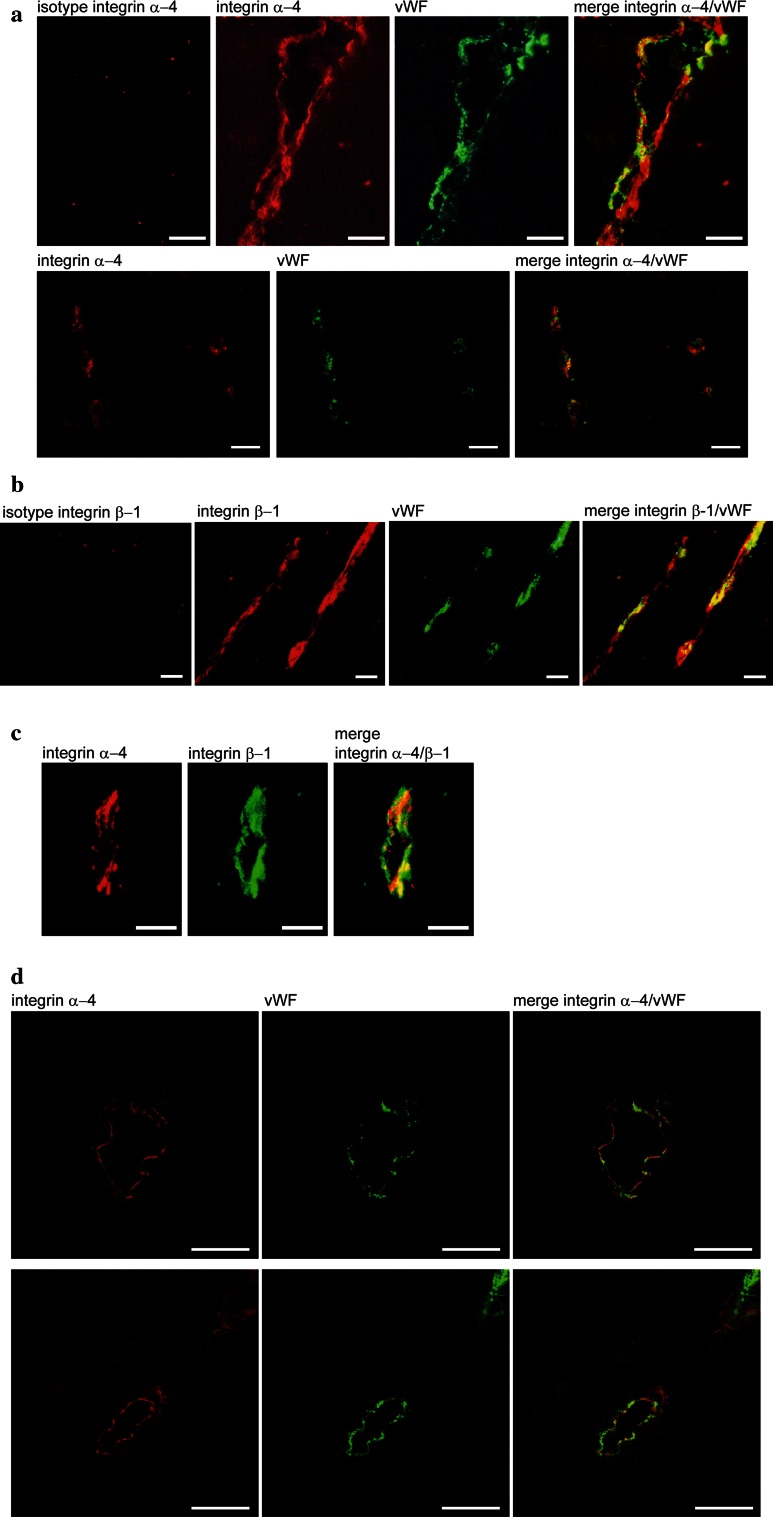
Human CNS microvascular endothelial cells show moderate integrin α-4 and strong β-1 expression in situ. Cryopreserved early post-mortem normal human brain tissue was double-stained for integrin α-4 (*red*) and for vWF (*green*) as an endothelial marker (**a**), integrin β-1 (*red*) and vWF (*green*) (**b**) or for integrin α-4 (*red*) and β-1 (*green*) (**c**). Representative for normal brain and spinal cord samples from three different donors. **d** To further study the subcellular localisation of integrin α-4, cryopreserved brain biopsy samples from two donors without evidence for pathological changes, as revealed by detailed neuropathological evaluation, were analysed by confocal microscopy. Integrin α-4 is shown in *red*, vWF in *green*. *Yellow* in the merge images indicates co-localisation of the investigated molecules. *Scale bars* 25 µm

Expression of integrin α-4 and its heterodimerisation partners β-1 and β-7 was previously described in various non-CNS human endothelial cell types [6, 26, 29]. In contrast, integrin α-4 expression was not previously reported in undiseased adult human brain endothelium. To investigate integrin expression by brain endothelial cells in situ, we performed immunohistochemical stainings on cryostat sections of early post-mortem normal human brain and spinal cord. Moderate integrin α-4 expression was detected in 310/400 (77.5 %) analysed vWF-positive blood vessels of various sizes in tissue samples from all three tested donors (examples shown in Fig. 1a). In contrast to only moderate, non-uniform integrin α-4 expression, strong endothelial β-1 expression was uniformly observed in all vWF-positive blood vessels of all donors (examples shown in Fig. 1b). Accordingly, all integrin α-4-positive vessels were β-1 positive (example shown in Fig. 1c). No endothelial integrin β-7 expression was detected in situ (data not shown).

To further study the subcellular localisation of integrin α-4 in human brain endothelium in situ, we next investigated cryopreserved brain biopsy specimens from two donors without pathological changes in their biopsies, as revealed by extensive neuropathological evaluation. The expression of integrin α-4 was found to be mainly restricted to the luminal membranes and weaker detectable in the abluminal membranes (Fig. 1d).

**Fig. 2:**
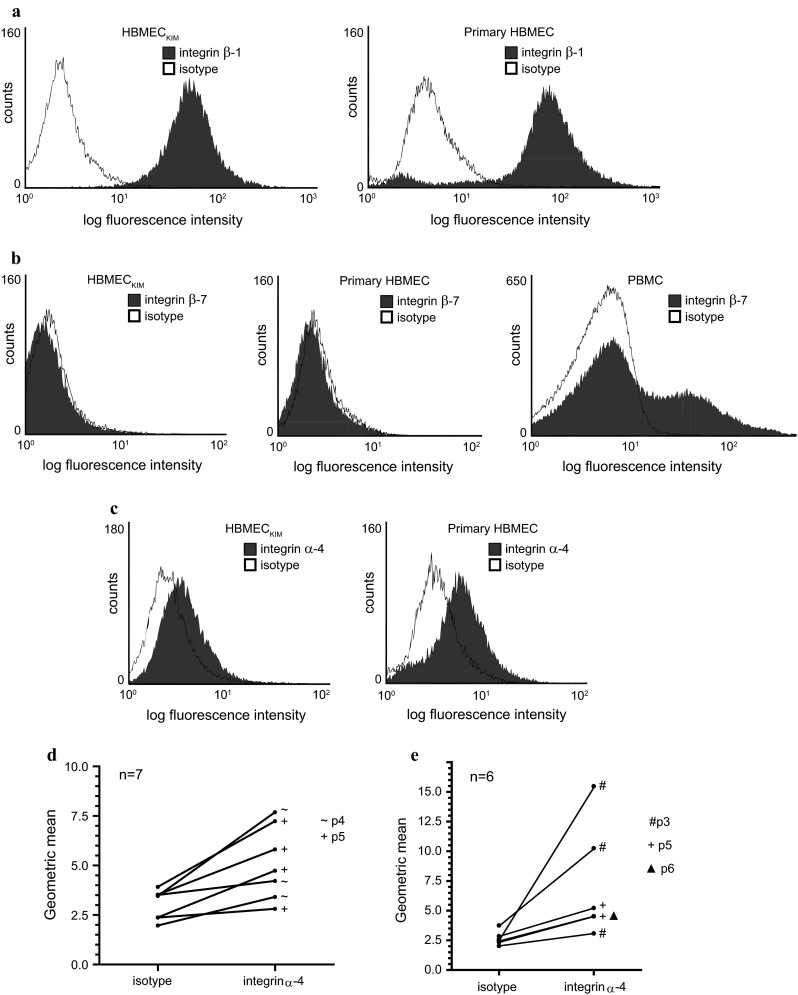
Brain endothelial cells show low to moderate integrin α-4 and strong β-1 but no β-7 expression in vitro. Flow cytometric analysis of integrin β-1 (**a**), β-7 (**b**) and α-4 (**c**) expression by the immortalised human brain endothelial cell line HBMEC_KIM_ and cultured single-donor primary cell preparations, or human PBMC as a positive control for integrin β-7 staining (shown in **b**). While integrin β-1 was strongly and permanently detected in all cell preparations, integrin α-4 was intermittently undetectable in the majority of preparations (see text); only positive examples shown in **c**. Note the different *X* axis labelling in **a** versus **b** and **c**. **d** Variability of integrin α-4 expression between different batches of an exemplary individual single-donor primary cell preparation under identical basic culture conditions as described in “Materials and methods” without previous starvation, forming a subconfluent monolayer (*n* = 7). Passage numbers are indicated as passage 4 or 5 (p4 or p5, respectively). **e** Variation of integrin α-4 expression levels between six different integrin α-4-positive single-donor primary cell preparations under identical basic culture conditions at different passages, referred to as p3, p5 and p6, respectively

To explore integrin expression on human brain endothelium in vitro, we next performed flow cytometric stainings of a well-characterised immortalised human brain microvascular endothelial cell line and of highly pure single-donor primary cell preparations, in particular the latter showing a well-preserved expression of tight junctions’ molecules and an intact paracellular barrier function [8, 21]. In line with the in situ stainings, strong permanent integrin β-1 and no β-7 expression were uniformly detected in all tested cell preparations (examples shown in Fig. 2a, b). Importantly, low to moderate integrin α-4 expression was detected in the immortalised cell line and 6/9 tested single-donor primary cell preparations (examples shown in Fig. 2c). Of note, integrin α-4 was not permanently expressed under basic culture conditions in vitro, but intermittently undetectable in the majority of positive preparations including the cell line. Integrin α-4 was found to be either detectable or undetectable after thawing and seeding of a cell vial but the status of integrin α-4 expression was usually found not to change during the same passage under non-stimulated conditions. In accordance with previous reports on other endothelial cell types [6, 32], integrin α-4 expression was generally lower than β-1 expression. Furthermore, it varied considerably between different batches of an individual single-donor primary cell preparation (Fig. 2d), however, not within single experiments, and it varied between different preparations (Fig. 2e). Both immunohistochemical detection of integrin α-4 on vWF-positive brain endothelium in situ and detection in the immortalised cell line provided evidence that integrin α-4 detection in the cultured primary cell preparations could at least not solely be attributed to potential contamination with other cell types, which was additionally excluded by extensive control stainings of each cell preparation as previously described [8]. Together, these in situ and in vitro experiments indicated low to moderate integrin α-4, strong β-1 and no β-7 expression by adult human brain endothelial cells in situ and in vitro.

### Increased endothelial integrin α-4 expression after angiogenic stimulation in vitro and in active MS brain lesions

**Fig. 3:**
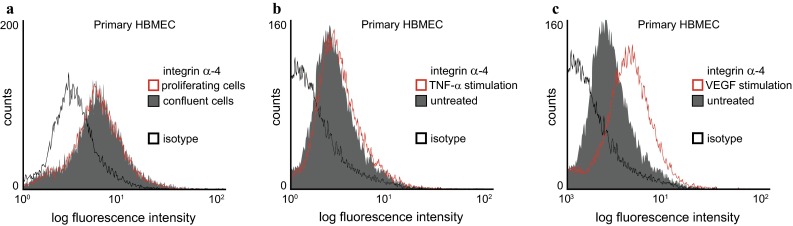
Upregulation of brain endothelial integrin α-4 expression after angiogenic stimulation in vitro. **a** Flow cytometric comparison of integrin α-4 expression on proliferating versus confluent primary human brain endothelial cells. Analysis of integrin α-4 expression after stimulation of subconfluent primary brain endothelium with 10 ng/mL TNF-α (**b**) or 10 ng/mL VEGF_165_ (**c**) for 24 h. Each condition representative of at least five independent experiments with different integrin α-4-positive primary brain endothelial cell preparations

In contrast to integrin β-1, the expression of integrin α-4 by cultured brain endothelium was found to be unstable within individual cell preparations. We therefore next sought to identify factors modulating its expression. A comparison of proliferating and confluent primary brain endothelial cells, which were cultured in parallel, did not reveal different integrin α-4 expression levels on the cell surface (Fig. 3a). As shown in Fig. 3b, pro-inflammatory stimulation with TNF-α did not reveal a significant effect on integrin α-4 expression either, which was in contrast to previous reports on other endothelial cell types, where an upregulation of integrin α-4 expression was observed after TNF-α stimulation [6, 32]. Stimulation with IFN-γ, IFN-β, IL-6 and TGF-β did not alter integrin α-4 expression either (data not shown). In contrast, stimulation with recombinant VEGF_165_, a variant of VEGF-A, induced a variable upregulation of integrin α-4 expression by a mean of 23 % (range +0.3 % [no effect in one single preparation] to +45.2 %, one example shown in Fig. 3c), being in line with previous reports where endothelial VLA-4 was found to be involved in angiogenesis [32, 41]. While integrin β-1 expression levels did not change after cytokine stimulation, integrin β-7 remained undetectable (data not shown). Although not formally analysed, increasing passage numbers did not systematically alter integrin α-4 expression levels in our observation. In summary, we identified VEGF as one cytokine upregulating the expression of integrin α-4 on cultured primary human brain endothelial cells.

**Fig. 4:**
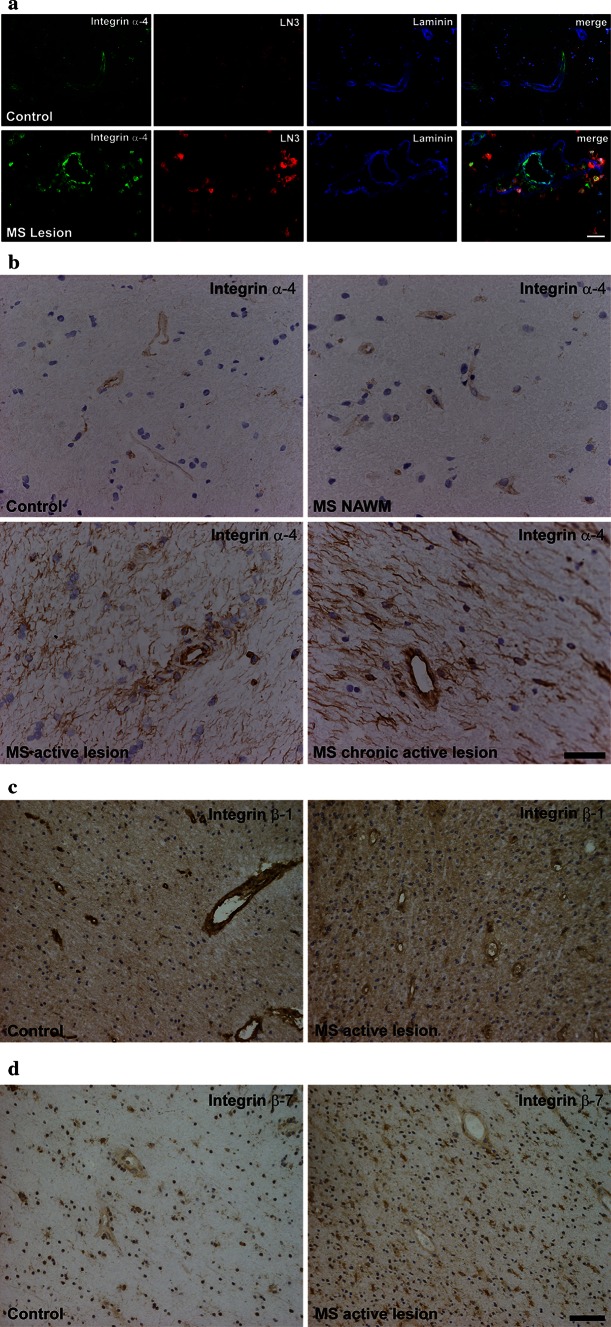
Expression of brain endothelial integrins α-4, β-1 and β-7 in human active white matter lesions in post-mortem material of MS cases compared to controls without clinical and autoptic evidence for a pathological CNS condition. MS lesion activity was characterised by myelin staining against proteolipid protein and against HLA-DR to visualise mononuclear immune cells and activated microglia (data not shown). **a** Immunoreactivity for integrin α-4 (CD49d) is labelled in *green*, HLA-DR (clone LN3) in *red* to visualise immune cells and laminin in *blue* to visualise the vascular basement membrane. The *right* *column* shows merged images. **b** DAB bright field stainings against integrin α-4 of samples from different cases than those shown in **a** to demonstrate tissue preservation, relation of blood vessels to the surrounding tissue and regulation of integrin α-4 expression. **c**, **d** Expression of integrin β-1 (**c**) or integrin β-7 (**d**) in active MS lesions versus control tissue, as demonstrated by DAB bright field stainings. Representative of autoptic samples from supratentorial white matter of seven different MS cases and four controls (1 tissue block per case). *Scale bars* 10 µm (**a**, **b**) or 50 µm (**c**, **d**)

VEGF is strongly expressed in inflammatory-active MS lesions [35, 40], its serum levels were found to be increased during relapses [44], and astrocyte-derived VEGF mediated a disruption of the BBB in animal models of the disease [3, 4]. We therefore next investigated endothelial integrin α-4 expression in MS brain lesions. A strong vascular expression of integrin α-4 was observed in inflammatory-active demyelinating lesions, whereas control tissue displayed only moderate immunoreactivity to integrin α-4 (Fig. 4a, b). In contrast, strong vascular expression of integrin β-1 was found to be downregulated in active MS lesions as compared to control tissue, although it remained clearly detectable in active MS lesions (Fig. 4c). While integrin β-7 had not been detectable in immunofluorescence stainings of normal human brain and in cultured brain endothelial cells as reported, very faint and diffuse anti-integrin β-7 DAB staining of vessel walls was observed both in control tissue and active MS lesions. These stainings did not provide definite evidence for an expression of integrin β-7 by brain endothelial cells in situ in our interpretation. In summary, endothelial integrin α-4 was upregulated while integrin β-1 was partially downregulated in inflammatory-active MS brain lesions as compared to control tissue.

### Soluble VCAM-1 increases brain endothelial permeability via integrin α-4 and alters actin and tight junction morphology

**Fig. 5:**
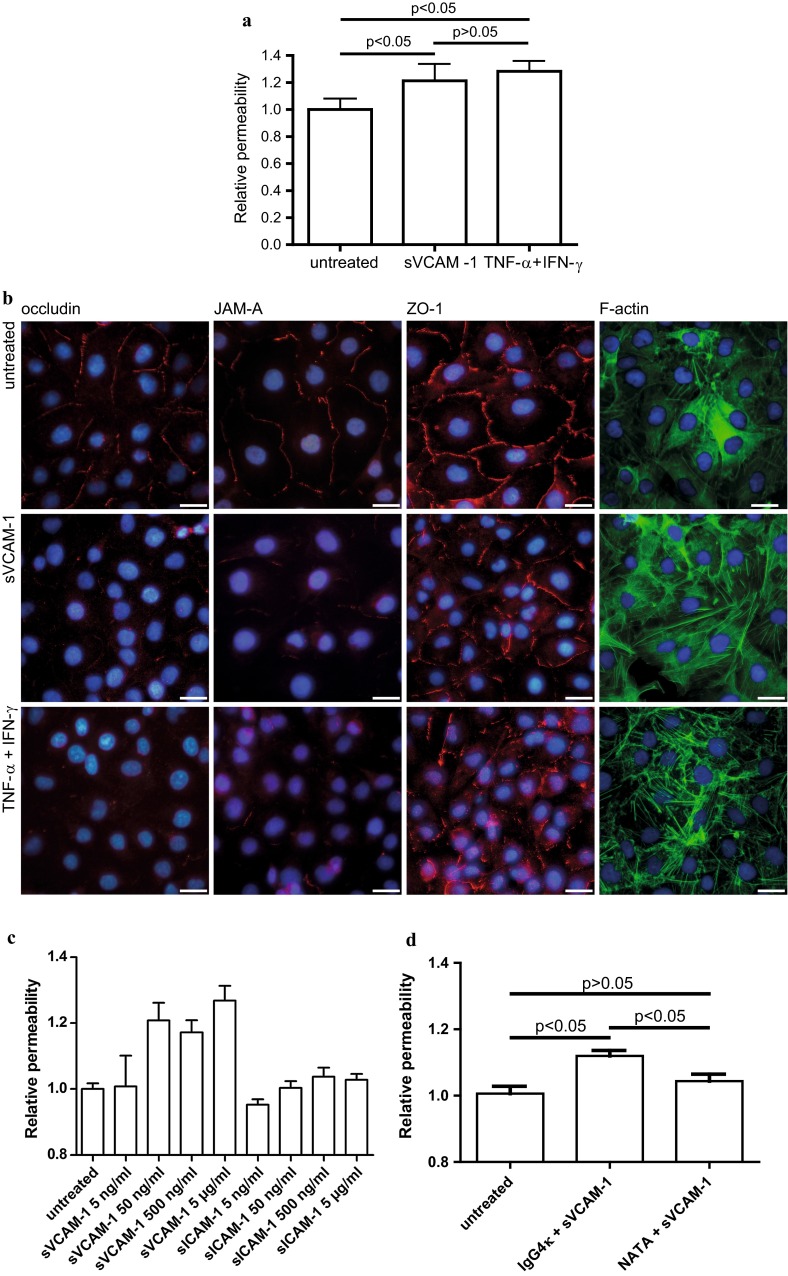
Soluble VCAM-1 increases paracellular permeability of brain endothelial cells, which is inhibited by natalizumab, and induces actin stress fibre formation and alters tight junction morphology. **a** Analysis of paracellular barrier function by dextran 3000 Boyden chamber assays. Stimulation with 5 µg/mL sVCAM-1, or 10 ng/mL TNF-α + 100 IU/mL IFN-γ as a positive control for 1 h. All stimulations were performed in dodecaplicates. Mean and SEM of four independent experiments with different integrin α-4-positive primary HBMEC preparations. Statistical analysis by Kruskal–Wallis test and Dunn’s post test for multiple comparisons. **b** Immunocytochemical staining of the tight junction-associated molecules occludin, JAM-A and ZO-1, and F-actin staining with Alexa Fluor 488 phalloidin after stimulation of primary HBMEC as in **a**. Representative of four independent experiments evaluated by blinded investigators. *Scale bar* 25 µm. **c** Concentration-dependent regulation of integrin α-4 expression after stimulation with sVCAM-1 or sICAM-1 at the indicated concentrations ranging from 5 to 5 µg/mL for 1 h. Mean and SEM of a Boyden chamber dextran permeability assay as described in **a** representative of three independent experiments. **d** Soluble VCAM-1-induced increase in paracellular permeability is partially inhibited by pre-incubation with natalizumab (NATA). Pre-incubation with 3 µg/mL of either natalizumab, a humanised monoclonal antibody against integrin α-4, or IgG4κ as the corresponding isotype control for 1 h was followed by addition of 5 µg/mL sVCAM-1 for 1 h. Mean and SEM of four independent Boyden chamber dextran permeability assay experiments as described in **a**. Note that *y* axis does not start from zero in **c** and **d**

Having studied the expression of sVCAM-1-binding integrins on adult brain endothelial cells, we next evaluated whether sVCAM-1 affects their paracellular barrier function. Boyden chamber permeability assays using dextran 3000 as a tracer revealed an increase of dextran permeability after stimulation of integrin α-4-positive primary brain endothelium with recombinant sVCAM-1 for 1 h (Fig. 5a). This increase of paracellular permeability was paralleled by a reorganisation of the actin cytoskeleton with formation of stress fibres. Furthermore, we found reduced cell border staining of the tight junction-associated proteins occludin, JAM-A and ZO-1 (Fig. 5b). All immunocytochemical stainings were evaluated by blinded investigators. Both sVCAM-mediated loss of barrier function and alteration of tight junction morphology principally resembled the pro-inflammatory action of TNF-α and IFN-γ, with particularly morphological changes of endothelial cells being less pronounced after sVCAM-1 stimulation. While sVCAM-1 exerted a barrier-disturbing effect already at a concentration of 50 ng/mL in our in vitro system, a 100-fold higher concentration of sICAM-1, another cell adhesion molecule of the immunoglobulin superfamily, did not alter endothelial barrier function (Fig. 5c). Importantly, blocking integrin α-4 by the clinically well-established humanised neutralising monoclonal Ab natalizumab partially inhibited the sVCAM-1-induced increase of paracellular permeability (Fig. 5d)—demonstrating that the barrier-disturbing effect of sVCAM-1 was at least partially mediated by integrin α-4 and suggesting a novel protective mode of action of natalizumab at the BBB.

### Rho GTPase and p38 mediate sVCAM-1-induced alteration of tight junction morphology

**Fig. 6:**
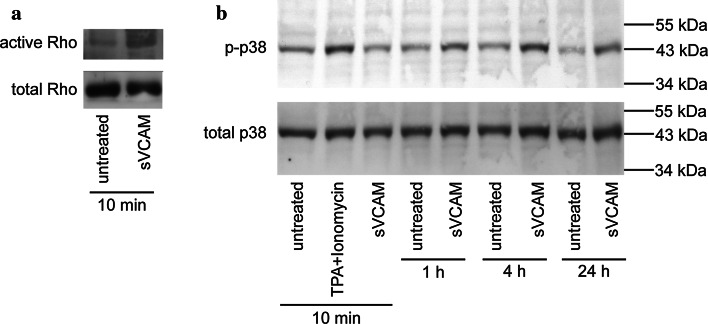
Soluble VCAM-1 activates Rho GTPase and p38 MAP kinase in brain endothelial cells. **a** Western blot analysis of Rho activation in integrin α-4-positive primary HBMEC after stimulation with 5 µg/mL sVCAM-1 for 10 min. **b** Western blot analysis of p38 MAP kinase activation after stimulation with 5 µg/mL sVCAM for the indicated durations. Stimulation with 12-*O*-tetradecanoylphorbol-13-acetate (TPA) and ionomycin for 10 min was used as a positive control. Representative of four independent experiments with different primary cell preparations

Based on reports that sVCAM-1 may induce VLA-4-mediated intracellular signalling events both in mononuclear cells [39] and in human endothelial cells [32], we next sought to identify intracellular signalling molecules activated by sVCAM-1 in brain endothelial cells. As actin stress fibres were induced by sVCAM-1, we chose Rho GTPase, which is critically involved in brain endothelial stress fibre formation [2, 28], as one molecule of interest. Based on a report about integrin α-4-transduced p38 MAP kinase activation in sVCAM-1-stimulated HUVEC [32], we furthermore studied activation of MAP kinases. We found rapid activation of Rho upon stimulation of primary HBMEC with sVCAM-1 (Fig. 6a). Furthermore, we observed a sustained activation of p38 MAP kinase (Fig. 6b) but not of ERK1/2 or JNK (data not shown). These experiments indicated rapid activation of Rho and sustained activation of p38 MAP kinase by sVCAM-1 in adult brain microvascular endothelial cells.

**Fig. 7:**
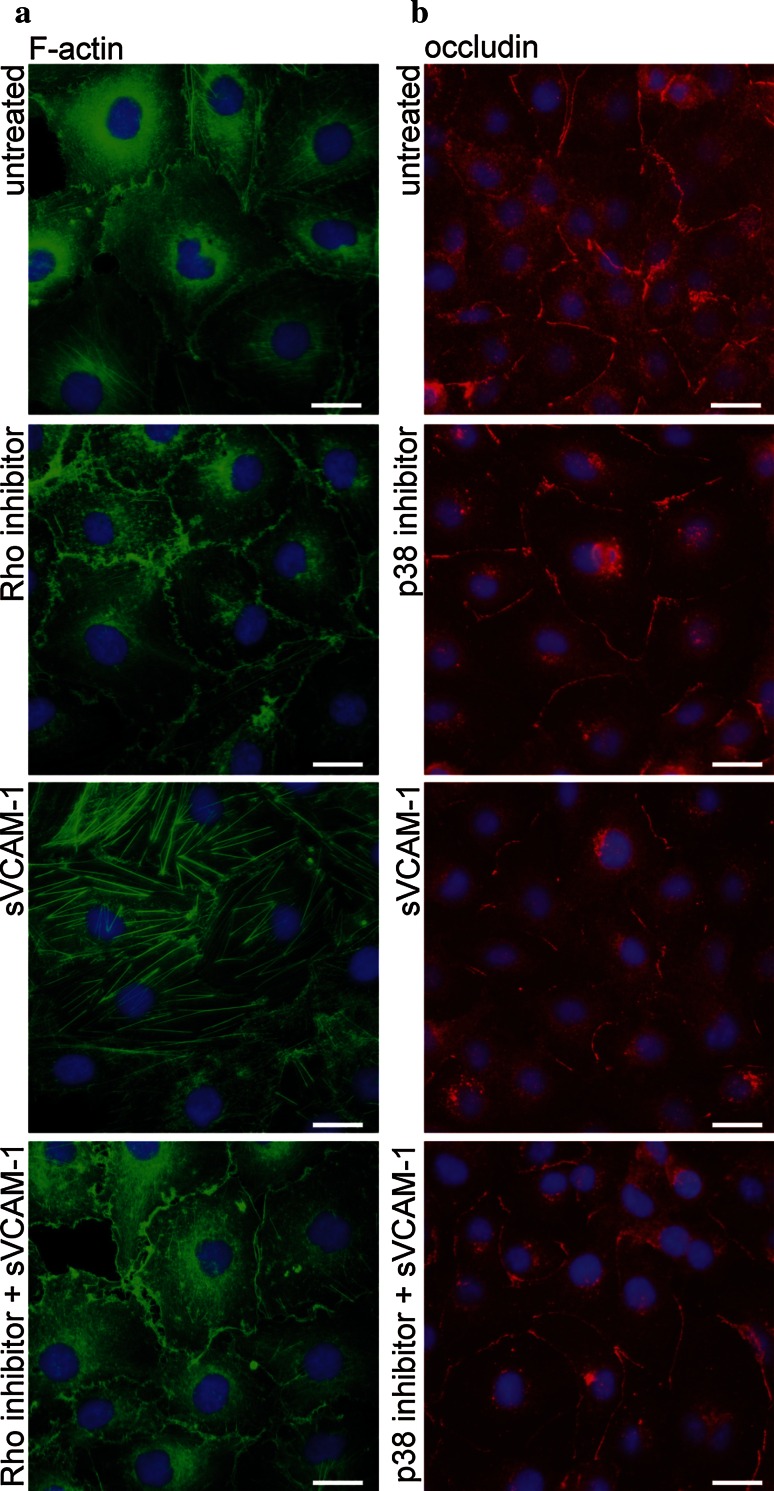
Effects of sVCAM-1 on tight junction morphology are partially mediated by Rho GTPase and p38 MAP kinase activation. Immunocytochemical analysis of F-actin (**a**) and occludin (**b**) expression in primary HBMEC which were left untreated, treated with 2 µM Rho-associated kinases inhibitor Y-27632 or 10 µM p38 inhibitor SB203580 for 2 h, 5 µg/mL sVCAM-1 for 1 h, or by one of the inhibitors for 2 h and additional presence of 5 µg/mL sVCAM-1 for the last hour. Representative of five independent experiments evaluated by blinded investigators. *Scale bar* 25 µm

In a last step, we investigated whether the identified intracellular signalling molecules mediated the barrier-disturbing effect of sVCAM-1. Chemical inhibition of Rho-associated kinases (ROCK) by Y-27632 or of p38 by SB203580 partially prevented sVCAM-1-induced actin reorganisation (Fig. 7a) and altered expression of tight junction-associated molecules in primary endothelial cells (Fig. 7b). These experiments indicated that sVCAM-1-induced tight junction reorganisation was partially mediated by the identified sVCAM-1-induced intracellular signalling events in primary human brain endothelium.

## Discussion

Here we report that non-inflamed adult human brain microvascular endothelium expresses integrin α-4 in situ and in vitro. Integrin α-4 was found to be upregulated after angiogenic stimulation in vitro and in active demyelinating MS brain lesions. Integrin α-4/β-1 is an established binding partner of sVCAM-1 which gets released from brain endothelial cells under inflammatory conditions [25]. In our hands, recombinant sVCAM-1 compromised brain endothelial barrier function in vitro, which was mediated by the induction of intracellular signalling events including the activation of Rho GTPase and p38 MAP kinase through binding of sVCAM-1 to integrin α-4. While the MS therapeutic natalizumab is well known to exert a protective effect at the BBB by blocking leukocyte adhesion, our findings suggest a novel additional protective mode of natalizumab action at the BBB by partially inhibiting integrin α-4 on brain endothelial cells.

Our in vitro findings indicate that a positive correlation between sVCAM-1 serum levels and Gd-enhancing MRI brain lesions in untreated MS patients, as observed by many authors [15, 18, 19, 22, 36, 37], at least partially reflect a direct causal relationship between both variables: sVCAM-1, which is released from inflammatory-activated human brain endothelium [24], could enhance BBB dysfunction in acute MS brain lesions by endothelial autocrine stimulation. Accordingly, increased sVCAM-1 serum levels in untreated MS patients with active disease would particularly be explained by a sVCAM-1 release from endothelium in inflammatory-active brain lesions. In contrast, an elevation of sVCAM-1 serum levels by IFN-β treatment could be due to a sVCAM-1 release particularly from vascular beds exposed to very high IFN-β concentrations, i.e. near the injection sites, but possibly not mainly the brain. However, brain endothelial cells were shown to release sVCAM-1 upon IFN-β exposure in vitro, establishing a direct causal link between exposure of endothelial cells to IFN-β and a release of sVCAM-1 [11, 25]. At IFN-β skin injection sites, strong inflammatory immune reactions, possibly boosting an IFN-β-induced release of sVCAM-1 from local endothelial cells, occur frequently. Such inflammatory reactions are common even if no externally visible skin reactions are present, as demonstrated by a placebo-controlled skin biopsy study [7]. Further following this explanatory model of a spatially differential sVCAM-1 release in untreated active versus IFN-β-treated MS patients, an inverse correlation between sVCAM-1 serum levels and Gd-enhancing MRI brain lesions in IFN-β-treated MS patients could then be explained by a predominant therapeutic effect of sVCAM-1-triggered VLA-4 downregulation on PBMC via ligand–receptor interaction as previously demonstrated [31]. This may render them less responsive to endothelium-bound VCAM-1 expressed in inflammatory-active MS brain lesions and therefore reduce inflammatory MRI disease activity. Importantly, VLA-4 downregulation on PBMC inversely correlated with both sVCAM-1 serum levels and with clinical treatment response [42]. Together, these findings argue for a predominant sVCAM-1 effect on immune as opposed to brain endothelial cells in IFN-β-treated patients. In summary, we suggest that sVCAM-1 may exert either a detrimental or a beneficial net effect on inflammatory MS disease activity, depending on the body region of its primary release. When released at an inflamed BBB where its endothelial-binding partner integrin α-4 is upregulated and integrin β-1 is present according to our study, it may further increase paracellular BBB dysfunction by endothelial autocrine stimulation, reflected by more Gd-enhancing MRI lesions. When released near IFN-β injection sites, sVCAM-1 may primarily render PBMC less responsive to endothelium-bound VCAM-1 in active MS brain lesions by VLA-4 downregulation on PBMC.

Integrin α-4, which was demonstrated to be expressed by endothelium in MS brain lesions in this study, is the molecular target of natalizumab. This humanised monoclonal IgG4κ antibody was approved for the treatment of severe relapsing–remitting MS in 2006. Therapy of MS patients with natalizumab reduced the number of Gd-enhancing MRI lesions by 92 % over 2 years in the AFFIRM trial [34]. This strong therapeutic effect of natalizumab most likely reflects its main mechanism of action in patients with MS, i.e. blockade of the molecular interaction between VLA-4 on T cells and VCAM-1 on the surface of brain endothelial cells, thereby strongly reducing inflammatory brain infiltrates [10]. Our findings, however, suggest that in addition natalizumab may beneficially modulate a potential detrimental interplay between integrin α-4/β-1 on brain endothelium and sVCAM-1 as suggested by this study.

In summary, we demonstrated that sVCAM-1 directly compromises the barrier function of human brain endothelium by integrin α-4/β-1-mediated induction of intracellular signalling events. Based on these findings, we suggested a model of how to explain apparently contradictory findings on the role of sVCAM-1 in untreated versus IFN-β-treated MS patients. Furthermore, our results argue for a novel mode of action of natalizumab at the BBB, where it may partially protect brain endothelial cells from a sVCAM-1-mediated barrier breakdown.

## Abbreviations

BBB: Blood–brain barrier
Gd: Gadolinium
HBMEC: Human brain microvascular endothelial cells
HUVEC: Human umbilical vein endothelial cells
IFN-β: Interferon β
MS: Multiple sclerosis
NATA: Natalizumab
PBMC: Peripheral blood mononuclear cells
sVCAM-1: Soluble vascular cell adhesion molecule-1
VLA-4: Very late antigen-4
